# The relationship between emotional intelligence and job satisfaction among nurses in Accra

**DOI:** 10.1002/nop2.70

**Published:** 2016-11-06

**Authors:** Theophilus Tagoe, Emmanuel Nii‐Boye Quarshie

**Affiliations:** ^1^ Department of PsychologyUniversity of GhanaLegonGhana; ^2^ School of PsychologyUniversity of LeedsLeedsUK

**Keywords:** Accra, emotional intelligence, Ghana, job satisfaction, nurses

## Abstract

**Aim:**

The aim of this study was to examine the relationship between emotional intelligence and job satisfaction among nurses in Accra, Ghana.

**Methods:**

A correlational study was conducted in Ghana in 2015. The study conveniently sampled 120 registered general nurses (83 females and 37 males) from three public hospitals located in Accra. The Schutte Self‐Report Emotional Intelligence Inventory and the Job Satisfaction Survey were used to assess emotional intelligence and job satisfaction respectively.

**Results:**

The findings showed a significant positive correlation between emotional intelligence and job satisfaction among the nurses. However, the results revealed no significant gender difference in emotional intelligence and job satisfaction.

## 1 Introduction

About 24% of the global disease burden is recorded in Africa, but only 3% of the world's healthcare workforce can be found on the continent to tackle it (World Health Organization, 2006). Thus, the African continent, especially sub‐Saharan Africa is faced with human resource crisis in healthcare delivery. The tremendous disease burden and frail health system in sub‐Saharan Africa are aggravated by the significant level of poverty, underdevelopment, conflict and poorly‐managed governmental institutions in the region (Anarfi, Quartey, & Agyei, 2010; Dovlo, 2007). The shortage of healthcare personnel in the region, precisely, nurses are as a result of ‘brain drain’ – the migration of professionals in the health sector (Dovlo, 2007; Martineau, Decker, & Bundred, 2004). This has been the case of most sub‐Saharan African countries such as Ghana. In the light of this, recruitment and retention of nurses have become issues of great concern to the government of Ghana. The government and stakeholders must adopt strategies to curb this situation and hence improve on working conditions of nurses. This would ensure that there is increasing number of nurses in the Ghana with high levels of job satisfaction (Snow et al., 2011).

Emotional intelligence is the ability to perceive emotions, integrate emotions to facilitate thoughts, understand emotions and to regulate emotions to promote personal growth (Mayer & Salovey, 1995). Emotional intelligence correlates with and somehow predicts job satisfaction (JS) among workers in certain settings. For instance, Lee and Ok (2012) found a positive correlation between emotional intelligence and job satisfaction among hospitality workers. They maintain that the significance of emotional intelligence cannot be disputed in the lives of workers in relation to job satisfaction. Emotional intelligence helps one to understand and manage emotions, therefore, helping workers to take control of their work (Lee & Ok, 2012).

According to Cekmecelioglu, Gunsel, and Ulutas (2012): ‘Job satisfaction is the conceptualization of personalistic assessment of conditions existing on the job or outcomes resulting from having a job’ (p. 364). Given the crucial roles played by nurses in the delivery of quality healthcare, it is imperative to ensure their job satisfaction. Pillay (2009) argues that it is important to comprehend what motivates nurses and the level to which organization and other contextual variables satisfy them. Hospital staff have to deal, usually on daily basis, with events bound with emotions such as births, illnesses, accidents and deaths. However, nurses are expected to manage such stressful situations, alongside the professional obligation to perform most effectively (Trivellas, Gerogiannis, & Svarna, 2013). Thus, it is prudent that nurses possess some psychological and emotional qualities (including emotional intelligence) to be able to manage and cope with such stressful situations and also perform effectively, because ‘high responsibility on patients’ treatment is of utmost importance’ (Trivellas et al., 2013 p.702): negligence; ignorance and improper attitude on the part of the nurses might cost loss of lives.

The relationship between emotional intelligence has gained the attention of researchers as it is reported that emotional intelligence plays a pivotal role in predicting job satisfaction among employees (Ghoreishi et al., 2014). In a recent study to examine the impact of emotional intelligence at the workplace on job satisfaction and turnover intentions of nursing staff working in hospitals, Trivellas et al. (2013) found that emotional intelligence exerts a significant impact on both job satisfaction and turnover intentions. Anari (2012) observed a positive significant relationship between emotional intelligence and both job satisfaction and organizational commitment. In relation to emotional intelligence, the findings provided support for gender difference, with females reporting higher scores on emotional intelligence than their male counterpart. However, the study found no significant gender difference and age difference on job satisfaction and organizational commitment. Similarly, Emdady and Bagheri (2013) found a high positive correlation between emotional intelligence and job satisfaction among employees in Sama organization in Iran. However, no statistically significant gender difference on job satisfaction was reported among the respondents (Emdady & Bagheri, 2013). The evidence is that, generally, in low‐and middle‐income countries, nurses tend to report higher levels of job satisfaction compared with other primary healthcare providers in the public sector (see Kumar, Khan, Inder, & Sharma, 2013).

There is a dearth of systematic research on the connection between emotional intelligence and job satisfaction among nurses in Ghana. Thus, this study is a seminal correlational study focused on examining the relationship between emotional intelligence and job satisfaction among nurses in Accra, Ghana. Furthermore, it is hoped that the findings of this study will contribute to the building of an evidence base on the area in Ghana. Based on the literature above, the study hypothesized that:

1. Emotional intelligence will have a positive correlation with job satisfaction among nurses.
2. There will be no significant gender difference on emotional intelligence scores among nurses.
3. Gender difference on job satisfaction will not be significant among nurses.

## 2 The study

### 2.1 Aim

This study sought to contribute to the evidence base of research output on the psychological well‐being of nursing professionals by examining the relationship (if any) between emotional intelligence and job satisfaction among nurses in Accra, Ghana.

### 2.2 Design

Based on the aim, a correlational design – ‘the collection of data to determine whether and to what degree, a relationship exists between two or more quantifiable variables’ – was employed for this study (Gay, Mills, & Airasian, 2011, p. 204).

### 2.3 Participants and setting

Ghana, located in the Western part of Sub‐Saharan Africa, was the setting for this study. Ghana's national nurse‐population ratio is 1:1251 (Netherlands Enterprise Agency, 2015), with 1:917 in respect of the Greater Accra region. The present and historical evolution of the training and educational system of nursing and nurses in Ghana have been thoroughly discussed elsewhere (see Opare & Mill, 2000; Talley, 2006). Consistently across time, nursing in Ghana has remained a female‐dominated profession, with relatively less number of men entering the profession (Kwansah et al., 2012; Talley, 2006). For example, a recent national‐level study has shown that the percentage of female‐male distribution of nurses in Ghana is 80:20 (Boafo, Hancock, & Gringart, 2016). Presently, there are about 11 cadres of nursing and midwifery personnel licensed by the Nursing and Midwifery Council of Ghana—NMCG (2016). These cadres include (but not exclusively limited to) Registered General Nurses (RGNs); Registered Midwives; Registered Community Nurses; Public Health Nurses; and Nurse Assistants (Clinical). RGNs are those who have undergone a 3‐year (diploma) and 4‐year (degree) course in nursing in accredited institutions (ibid). Relatively, RGNs are the preponderant cadre of nurses in Ghana. They are mainly trained and employed by government and posted to primary, secondary and tertiary healthcare facilities across the country. RGNs in Ghana are in charge of all general nursing duties – from out‐patient units to emergency departments – in the clinical setting on daily basis and are sometimes the only category of nurses (apart from midwives) found in some primary medical facilities in Ghana (Kwansah et al., 2012; Talley, 2006).

Accra (the principal city of the Greater Accra region) which doubles as the national capital of Ghana was the exact data collection site for this study. The population for this study consisted of RGNs in three public hospitals in Accra: two secondary hospitals and one tertiary hospital. These hospitals were deemed appropriate because compared with other hospitals in the country; they have a larger healthcare workforce with diverse backgrounds and orientation. Also, the hospitals are public facilities that admit people from all walks of life requiring employees to be sensitive to them. The convenience sampling technique was used to recruit 120 (i.e. 83 female & 37 male) nurses within the age bracket of 20–60 years for the study. The convenience sampling technique was deemed appropriate because it allowed for the selection of participants who were available at the particular point in time of the study and who were willing to respond to the survey (McLeod, 2014). Some nurses approached by the researchers declined to participate mainly due to their busy work schedule and sometimes unpredictable call of duty associated with their daily nursing duties.

### 2.4 Inclusion and exclusion criteria

An available nurse was recruited to participate if she/he had been in active nursing practice as a RGN for, at least, the past 12 months before the survey. However, an available nurse was denied participation if she/he had been in active nursing practice for less than 12 months prior to the survey and/or was of a different nursing category other than RGN, for example, midwifery, psychiatric nursing, *inter alia*. Cohen (1992) reported that the sample sizes necessary for 0.80 power at 0.05 level of significance for Pearson *r* is 85 and for independent *t*‐test is 64. Thus, the sample size adopted for this study was ideal, per this criterion by Cohen (1992).

### 2.5 Data collections

Questionnaires were used to collect the data. The questionnaires consisted of three sections. Section A gathered information on the respondents’ demographic characteristics such as sex, age and level of education and, sections B and C consisted of measures of emotional intelligence and job satisfaction, respectively.

The Schutte Self‐Report Emotional Intelligence Inventory developed by Schutte et al. (1998) was used to measure emotional intelligence. This is a 33‐item scale. Some of the items on the scale include ‘I know when to speak about my personal problems to others’. According to Ciarrochi, Forgas, and Mayer (2006), ‘The internal and test–retest reliabilities of the SSRI total score are high, α_s _= .93 and .73, respectively’ (p. 38). The responses on the scale were rated on 5‐point scale ranging from 1 = strongly disagree to 5 = strongly agree. The total scores on the scale range from 33 to 165, with higher scores reflecting higher levels of emotional intelligence.

The Job Satisfaction Survey (JSS) developed by Spector (1997) was used to measure the construct, job satisfaction. It contains 36 items measuring various facets of work. The JSS scale has an internal consistency of 0.91 for the total scale. A test–retest reliability of 0.71 was reported. The responses are rated on a 6‐point scale ranging from 1 = disagree very much to 6 = agree very much. The possible total scores on the scale ranges from 36 to 216 with the 36–108 range meaning dissatisfaction; 144–216 range meaning satisfaction; and between 108–144 depicting ambivalence.

Previous application of the Schutte Self‐Report Emotional Intelligence Inventory and the Job Satisfaction Survey in studies among education, healthcare and banking professionals in sub‐Saharan Africa (including Ghana) and other non‐Western countries have shown fair to strong reliability (e.g, Adeyemo, 2007, 2008; Al‐Faouri, Al‐Ali, & Al‐Shorman, 2014; Danquah, 2014; Hosseinian, Yazdi, Zahraie, & Fathi‐Ashtiani, 2008; Mousavi, Yarmohammadi, Nosrat, & Tarasi, 2012; Opuni & Adu‐Gyamfi, 2014). In this study, nurses were approached in their hospitals of work and at their respective nurses’ station where they responded to the questionnaire. Data were collected in the month of January, 2015.

### 2.6 Ethical considerations

Approval of the research protocol and ethical clearance for the study were obtained from the Department of Psychology, University of Ghana, Legon. Institutional permission to proceed with data collection was granted by the various administrative heads of the selected hospitals. After thoroughly informing the participants about the research, the researchers obtained both written and verbal consent from the participants, as some participants (1.6%) were unwilling to sign the actual consent forms. Participants were assured of anonymity and no item on the questionnaire solicited any direct identity information.

### 2.7 Data analysis

The statistical package for social science (IBM SPSS statistics base 20. Chicago, IL, USA: SPSS Inc.) was used for the analysis of the data. The researchers made use of both the descriptive and inferential statistical tools relevant to the data. The first hypothesis was tested using the Pearson Product‐Moment Correlation (Pearson *r*) as it sought to predict a relationship between two variables; and hypotheses 2 and 3 were analysed using the independent *t*‐test. This was because, essentially, each of the two hypotheses sought to compare the means of two independent samples.

## 3 Results

### 3.1 Sociodemographic characteristics

A total of 120 registered general nurses responded to the survey. As shown in Table 1, about 69.2% of the participating nurses were females; 76.7% of the participants were early adults (aged between 20–40 years old) with educational qualification ranging from nurses’ training college certificate through University degrees.

**Table 1:** Socio‐demographic characteristics of the sample frequencies and percentages

Variable	Category	Frequency (*n*)	Percentage (%)
Sex	Male	37	30.8
	Female	83	69.2
	Total	120	100
Age			
Early adults	[20–40 years]	92	76.7
Middle adults	[41–60 years]	28	23.3
Educational level	Certificate	13	10.8
	Diploma	53	44.2
	Bachelor's degree	51	42.5
	Master's degree	3	2.5
	Total	120	100

As evident in previous studies involving nurses in Ghana and other parts of Africa (e.g. Boafo et al., 2016; Kwansah et al., 2012; Sikiru & Shmaila, 2009), there were more female nurses in this study than male nurses. This is owed to the general fact that nursing is perceived as a female profession (Williams, 1992, 1995).

### 3.2 Hypotheses testing:

Hypothesis 1*: Emotional intelligence would have a positive correlation with job satisfaction among nurses*.

Results of the descriptive analysis and inferential testing of hypothesis 1 are shown in Table 2.

**Table 2:** Summary of the correlation between emotional intelligence and job satisfaction

Variable	Mean	Standard deviation	*N*	*r*
Emotional intelligence	125.06	14.02		
Job satisfaction	145.61	20.13	120	.398^a^

a *p* < .000.

The observed Pearson correlation coefficient (*r*) indicated a significant positive correlation between emotional intelligence and job satisfaction at 0.05 alpha level (*r*_ _ = .398, *p* < .05), thus confirming hypothesis 1.

Hypothesis 2: *There will be no significant gender difference on emotional intelligence scores among nurses*.

The observed *t*‐test indicated no significant difference between female nurses (mean = 125.30, *SD* 12.27) and male nurses (mean = 124.51, *SD* 17.50) in relation to scores on emotional intelligence [*t*_(118)_ = −0.283, *p *> .05]. This supported hypothesis 2.

Hypothesis 3: *Gender difference on job satisfaction will not be significant among nurses*.

The observed *t*‐test indicated no significant statistical difference between female nurses (mean = 146.67, *SD* 21.02) and male nurses (mean = 143.21, *SD* 18.02) on job satisfaction scores [*t*_(118)_ = −0.868, *p* > .05], confirming hypothesis 3.

## 4 Discussion

This study sets out to investigate the relationship (if any) between emotional intelligence and job satisfaction among nurses. Statistical analysis of the data shows three principal findings regarding nurses in Accra, Ghana: (1) a significant positive correlation exists between emotional intelligence and job satisfaction; (2) no significant gender difference exists in scores on emotional intelligence and (3) there is no significant statistical difference between females and males in terms of job satisfaction scores.

The first observation that a significant positive correlation exists between emotional intelligence and job satisfaction lends support to previous evidence in the area (e.g. Cekmecelioglu et al., 2012; Emdady & Bagheri, 2013; Lee & Ok, 2012; Mousavi et al., 2012; Trivellas et al., 2013). Nurses are generally trained and socialized to interact with their patients in cooperative and supportive ways, regardless of how demanding, challenging or even traumatic the nurse–patient circumstances may be (Seada & Fathi Sleem, 2012; Talley, 2006). Nurses tend to exhibit self‐awareness, self‐management, social‐awareness and relationship management, which pair up to form the personal and social competencies which make one emotionally intelligent (Anari, 2012; Bradberry & Greaves, 2005). Thus, it can be argued that, for an effective and successful nursing, the nursing professional has to be able to perceive, understand, regulate and harness their emotions (Schutte, Malouff, Simunek, McKenley, & Hollander, 2002). In this vein, job satisfaction appears somewhat consequential to emotional intelligence. Therefore, a nurse who deploys an appreciable degree of emotional intelligence in the performances of his/her job is also likely to report higher levels of job satisfaction on the job.

The second key finding that no significant gender difference was reported in scores on emotional intelligence in this study is consistent with previous evidence in the area (e.g. Anari, 2012; Emdady & Bagheri, 2013). Similarly, this evidence may be due to the professional socialization and training of nurses through which they acquire such professional values such as self‐esteem, ethical confidence and the value of empathizing with their patients (Butts & Rich, 2012; Iacobucci, Daly, Lindell, & Griffin, 2013; Seada & Fathi Sleem, 2012). Thus, nurses, irrespective of their gender, (at least, are ethically required to) place some premium on their professional nursing values (including those values related to emotion regulation, self‐esteem and emotional intelligence) in the context of nursing practice and thus are able to accurately appraise and express their emotions through the use of verbal and nonverbal competencies (Butts & Rich, 2012; Salovey & Mayer, 1990; Seada & Fathi Sleem, 2012). This possibly leads to the suppression of any potentially measurable gender difference in the professional nursing values (including emotional intelligence), they bring to bear in the performance of their duties as nurses.

Finally, consistent with previous findings (e.g. Emdady & Bagheri, 2013; Mabekoje, 2009), this study shows no significant difference between female and male on job satisfaction scores. Consistent with observations in other non‐Western and African contexts (see Boafo et al., 2016; Sikiru & Shmaila, 2009; Talley, 2006), more women tend to pursue the nursing profession and as such in a study like this, it is to be expected that significantly more female nurses than males will participate. Thus, it will not be unusual to expect females to score significantly higher on the factor of job satisfaction than their male counterpart as nursing is predominantly a female profession. However, the lack of any such gender difference (skewed in favour of female nurses) in this study could be largely due to what has been described as the ‘structural view’ of job satisfaction between women and men (Mason, 1995). The structural view of job satisfaction posits that women and men do not vary in terms of scores on job satisfaction and thus any observed gender difference on job satisfaction is attributable to other factors which co‐vary systematically with gender due to the prevalent segmentation of jobs based on gender in organizations (Gutek, 1988; Mason, 1995). This view appears tenable in the context of nursing in that although nursing is a female‐dominated profession, both male and female nurses are given equivalent opportunity structures (Laschinger, 1996; Williams, 1992, 1995). The implication is that both male and female nurses tend to have similar perception of the various facets of the nursing profession and job‐content resulting in similar scores on job satisfaction across gender.

### 4.1 Limitations

Although this study provides a seminal basis for the furtherance of research in the area emotional intelligence among nurses (and healthcare workers, more generally) in Ghana, it is limited in some ways. This study failed to explore which facets of job the participants deem important and thus contributing to their overall job satisfaction. In addition, the study did not focus on how well the participants fared on the various components of emotional intelligence and other factors that could moderate or mediate such relationships. It is therefore, suggested that future work should look at the various components of emotional intelligence and job facets and some moderating and mediating variables. Future studies of this kind could increase the sample size for the study to have higher external validity.

### 4.2 Conclusion

Drawing on the evidence established in this study, it can be concluded that nurses’ scores on emotional intelligence tend to be positively correlated with their scores on job satisfaction. However, emotional intelligence and job satisfaction do not have significant independent co‐variation relationship with gender. Further studies are required to expand the burgeoning evidence base of the relationship between emotional intelligence and other work‐related psychological variables among nurses in Ghana.

## Conflict of interest

No conflict of interest has been declared by the authors.

## Author contributions

All authors have agreed on the final version and meet at least one of the following criteria [recommended by the ICMJE (http://www.icmje.org/recommendations/)]:

- substantial contributions to conception and design, acquisition of data or analysis and interpretation of data;
- drafting the article or revising it critically for important intellectual content.
